# Cell-based, bioluminescent assay for monitoring the interaction between PCSK9 and the LDL receptor

**DOI:** 10.1194/jlr.D074658

**Published:** 2017-06-13

**Authors:** Sarah J. Duellman, Thomas Machleidt, James J. Cali, Jolanta Vidugiriene

**Affiliations:** Promega Corporation, Madison, WI 53711

**Keywords:** luminescence, cholesterol, antibodies, drug therapy, low density lipoprotein, proprotein convertase subtilisin/kexin type 9

## Abstract

Monitoring the expression of cell-surface receptors, their interaction with extracellular ligands, and their fate upon ligand binding is important for understanding receptor function and developing new therapies. We describe a cell-based method that utilizes bioluminescent protein complementation technology to interrogate binding of a cellular receptor with its extracellular protein ligand, specifically LDL receptor (LDLR) and proprotein convertase subtilisin/kexin type 9 (PCSK9). Purified, full-length tagged PCSK9 is added to assay wells containing cells that stably express LDLR with an extracellular complementary tag. When the tagged PCSK9 binds the receptor, a bright luminescence signal is generated. The interaction is detected at the cell membrane with add-and-read simplicity, no wash steps, and flexibility, allowing data to be collected in endpoint format, kinetically, or with bioluminescent imaging. The assay is flexible, is rapid, and reports accurate biology. It is amenable to 96-well and 384-well formats, and the robustness allows for screening of new drug candidates (*Z*′ = 0.83). The assay reports correct potencies for antibody titrations across a 50%–150% potency range and detects potency changes due to heat stress, suggesting that it may be useful during drug development. This assay technology can be broadly applied when studying other receptors with their extracellular ligands, whether protein or small-molecule binding partners.

The interplay between cell-surface receptors and their extracellular ligands is critical for understanding many biological systems and disease states. An example of the importance of these interactions is represented by the LDL receptor (LDLR) and proprotein convertase subtilisin/kexin type 9 (PCSK9) (1–4). The interaction of these two proteins affects the level of circulating LDL. Through binding to LDLR, PCSK9 targets LDLR for degradation leading to reduced surface receptor number and increased circulating LDL (5, 6). In the absence of PCSK9, the rate of LDLR recycling to the cell surface is increased, leading to enhanced receptor-mediated LDL clearance and reduced circulating LDL. Because of its role in regulating circulating LDL levels, PCSK9 has become an attractive drug target. Various approaches have been applied in attempts to lower serum LDL by blocking PCSK9 binding to the LDLR (7). The most advanced approach uses monoclonal antibodies (mAbs) that inhibit the LDLR-PCSK9 interaction. Inhibitory peptides or monobodies (adnectins) have also been used (8, 9). Two mAbs, alirocumab, (10, 11) and evolocumab (12, 13) were approved for therapeutic use as cholesterol-lowering drugs. Both drugs bind to PCSK9 and block its interaction with LDLR and are showing great promise in the clinic (14).

We describe a cell-based PCSK9-LDLR binding assay that monitors the interaction between a full-length LDLR fused to a luciferase fragment and full-length PCSK9 fused to a short peptide that complements the activity of the luciferase fragment. These fusion proteins use a protein-fragment complementation methodology based on NanoLuc luciferase (15, 16) to enable a luminescent cell-based assay. NanoLuc is a small, bright, and stable luciferase enzyme that generates a luminescence signal that is 100-fold brighter than firefly or renilla luciferase. These properties allow for substantial improvement in a number of luminescence applications, including the protein complementation methodology called NanoBiT (Nanoluc binary technology) that we used here to configure the PCSK9-LDLR binding assay. The method involves two protein fragments evolved from NanoLuc that are optimized for analysis of protein interactions: large BiT (LgBiT; 18 kDa) and small BiT (SmBiT; 1 kDa). The fragments form a bright luminescent enzyme only when bound together in a complementary fashion. However, these subunits have weak affinity on their own but are brought together by higher-affinity fusion partners. The association of LgBiT and SmBiT is reversible. In this way the NanoBiT fragments are ideal tags for monitoring protein-protein interactions. Protein interaction dynamics can be analyzed at a particular endpoint or monitored in real time in living cells following addition of the luciferase substrate. The assay was designed to analyze the interaction of these proteins at the cell surface.

We demonstrate that the PCSK9-LDLR binding assay is responsive to anti-PCSK9 antibodies that block the interaction. The assay is also suitable for high-throughput screening. The assay characteristics support drug development by detecting changes in stability of a drug target as well as changes in potency across a range of concentrations. This assay provides numerous advantages over current biochemical methods, including real-time kinetic analysis of the interaction in a cell-based system. Because the assay is cell based, it allows other factors that affect the interaction or fate of the receptor to be studied to determine how they influence the kinetics of binding. Many of these factors may yet be unknown and would be lost in an artificial, biochemical setting. Using the full-length proteins provides higher probability for detecting these contributions as well as influences that posttranslational modifications and expression levels would have on the interaction and kinetics.

## MATERIALS AND METHODS

### Reagents and equipment

All chemical reagents were obtained from Sigma-Aldrich unless otherwise specified. Anti-PCSK9 neutralizing antibody was from BPS Biosciences (San Diego, CA). Alirocumab was from Sanofi (Paris, France) and Regeneron Pharmaceuticals, Inc. (Tarrytown, NY). Evolocumab was from Amgen (Thousand Oaks, CA). Trastuzumab was from Genentech (South San Francisco, CA). All luminescence readings were performed on a Glomax Discover Detection System (Promega Corp., Madison, WI). EC_50_ calculations were performed using GraphPad Prism (version 6.03).

### Assay development

#### Generation of stable cell lines.

The full-length coding sequence for LDLR was cloned into the pNB3K NanoBiT cloning vector (Promega) to yield the fusion protein LgBiT-GSSGGGGSGGGGSSG-AIA-LDLR. The native LDLR signal sequence was removed and replaced with the interleukin-6 signal sequence (amino acid sequence: MNSFSTSAFGPVAFSLGLLLVLPAAFPAP). The full-length coding sequence for PCSK9 was cloned into the pNB2K NanoBiT cloning vector (Promega) to yield the fusion protein PCSK9-VSQ-GSSGGGGSGGGGSSG-SmBiT-Histag. HEK293 transfection was carried out using FuGENE® HD Transfection Reagent (Promega) at a lipid:DNA ratio of 3:1. After 48 h, media with transfection reagent were removed and replaced with DMEM media (Thermo Fisher Scientific) containing 10% FBS, penicillin/streptomycin, and 400 µg/ml G418 (Promega). A pool of cells stably transfected with LgBiT-LDLR were selected that gave a high signal in the PCSK9-LDLR binding assay. The LgBiT-LDLR cells were subjected to fluorescence-activated cell sorting analysis to interrogate the integrity of the receptor. The LgBiT-LDLR was labeled with anti-LDLR antibody, which suggests that the receptor localized to the cell membrane and displayed as expected. A pool of cells stably transfected with PCSK9-SmBiT were selected and maintained. Stability of both stable cell pools was confirmed to 20 passages.

### Expression and purification of PCSK9-SmBiT from mammalian cells

Media were collected from the cells that secrete PCSK9-SmBiT protein and clarified by centrifugation. PCSK9-SmBiT purification was performed on an AKTA PrimePlus purification system using buffer A (50 mM Tris-HCl pH 7.5, 500 mM NaCl, 0.1 mM CaCl_2_, and 25 mM imidazole) and buffer B (50 mM Tris-HCl pH 7.5, 500 mM NaCl, 0.1 mM CaCl_2_, and 500 mM imidazole). A HisTrap FF 1-ml column (GE Healthcare) was equilibrated in 100% buffer A. The media sample was spiked with reagent to bring the solution to 250 mM NaCl, 10 mM imidazole, and 50 mM Tris-HCl pH 7.5, then loaded directly onto the column. The column was washed with four column volumes (CV) of 100% buffer A, and a gradient elution was performed over 10 CVs from 0% to 100% buffer B. Purified protein was analyzed by gel electrophoresis, and peak fractions were combined and dialyzed overnight in dialysis buffer (25 mM Hepes pH 8.0, 150 mM NaCl, 0.1 mM CaCl_2_, and 10% glycerol). The SDS-PAGE gel confirmed that the PCSK9 protein migrated at the expected size, and the prodomain was cleaved.

### Assay performance

#### Thaw-and-use frozen cells.

HEK293 cells stably expressing LgBiT-LDLR were frozen at −80°C in 90% DMEM complete media and 10% DMSO. Cells were thawed, resuspended in Opti-MEM, and centrifuged. The supernatant was decanted and the cells were resuspended in Opti-MEM and immediately plated at 20,000 cells/well. At each time point, purified PCSK9-SmBiT was added (final concentration 0.8 μg/ml) and either Opti-MEM or alirocumab (final concentration 40 nM). This PCSK9 concentration is comparable to endogenous levels (17). Next, Nano-Glo Live Cell Substrate was added, following the manufacturer’s instructions (Promega), and luminescence was measured after a 15 min incubation.

### Endpoint format: Response to antibodies

LgBiT-LDLR-expressing HEK293 cells were plated at 10,000 cells/well in a 96-well plate in 100 µl of DMEM complete media (containing 10% FBS), and incubated in a cell culture incubator overnight. Then the medium was removed and replaced with 25 µl of Opti-MEM (Thermo Fisher Scientific), containing 2× PCSK9-SmBiT for a final concentration of 0.8 µg/ml and 25 µl of Opti-MEM containing a 2× antibody titration. Alirocumab and evolocumab were tested starting at 60 nM, and anti-PCSK9 neutralizing antibody (BPS Biosciences) and nonspecific control antibody, trastuzumab, starting at 20 nM. The reaction was incubated for 1 h; then 12.5 µl of 5× Nano-Glo Luciferase Assay Substrate (Promega) diluted into Opti-MEM was added to the reaction. The luminescence was measured after a 15 min incubation.

### Assay kinetics

LgBiT-LDLR-expressing HEK293 cells were plated at 10,000 cells/well in a 96-well plate in 100 µl of DMEM complete media (containing 10% FBS) and incubated in a cell culture incubator (37°C, 5% CO_2_, humidified atmosphere) overnight. Then the medium was removed and replaced with Nano-Glo Luciferase Assay Substrate diluted into Opti-MEM. To a subset of wells, Opti-MEM was added that contained PCSK9-SmBiT, for a final concentration of 0.8 µg/ml; other wells received Opti-MEM only for NanoGlo substrate background analysis. Luminescence was monitored every minute for 30 min. Then anti-PCSK9 neutralizing antibody (BPS Biosciences) was spiked into a subset of wells, for a final concentration of 15 nM, and luminescence readings were resumed.

### Bioluminescence imaging

HEK293 cells stably expressing LgBiT-LDLR were plated at a density of 40,000 cells/well in 400 μl growth medium in a Labtek chambered coverslip (Nunc). Following 24 h of incubation, the growth medium was removed by aspiration and replaced with 100 μl of Opti-MEM containing 15 nM PCSK9-SmBiT and either 30 nM anti-PCSK9 antibody (BPS Biosciences) or 75 nM nonspecific control antibody (trastuzumab), followed by 60 min incubation at 37°C in a tissue culture incubator. Immediately before imaging, 25 μl of 5× Nano-Glo Luciferase Assay reagent was added to each sample. Imaging was performed by using the Olympus LV200 bioluminescence imager, which is equipped with a Hamamatsu ImagEM CCD camera, a 40×/0.9 NA objective, and a temperature-controlled stage. Images were acquired with the acquisition feature of the Olympus CellSense software package. For image acquisition, exposure times/EM (electron-multiplying) gains were set to 50 ms/0 and 15 s/1000 for the brightfield and luminescence channels, respectively. Postacquisition processing was performed with the FIJI-ImageJ package. Each bioluminescence image was generated using an average intensity *Z* projection of an image stack containing 15 subsequently taken images.

### Analysis of the binding event

LgBiT-LDLR-expressing HEK293 cells were plated at 10,000 cells/well in a 96-well plate in 100 µl of DMEM complete media (containing 10% FBS) and incubated in a cell culture incubator (37°C, 5% CO_2_, humidified atmosphere) overnight. Then the medium was removed and replaced with either PCSK9-SmBiT (0.8 µg/ml final concentration, 15 nM) or a high-affinity complementation peptide 86 (15) (50 nM final concentration) in Opti-MEM. The high-affinity peptide 86 binds to LgBiT spontaneously with a K_D_ of 700 pM, leading to productive complementation of an active luciferase enzyme in the absence of facilitating protein partners. Then either 0 or 25 µg/ml (11 µM) LDL was added in Opti-MEM, and the reaction was incubated for 45 min at room temperature. The Nano-Glo Live Cell Substrate was added and luminescence measured after a 15 min incubation at room temperature.

LgBiT-LDLR-expressing HEK293 cells were plated at 20,000 cells/well in a 96-well plate in 100 µl of DMEM complete media (containing 10% FBS) and incubated in a cell culture incubator (37°C, 5% CO_2_, humidified atmosphere) overnight. The plate was equilibrated to 4°C, and then the medium was removed and replaced with cooled OptiMEM containing 1 μg/ml PCSK9-SmBiT and incubated at 4°C for 1 h. Wells were washed with OptiMEM and then replaced with fresh OptiMEM without PCSK9. A subset of wells contained dynole 34-2 endocytosis inhibitor (Tocris Bioscience). Nano-Glo Live Cell Substrate was prepared according to the manufacturer’s instructions, and luminescence was measured every 2 min for 100 min.

### Feasibility for high-throughput screening

LgBiT-LDLR HEK293 cells were plated at 20,000 cells/well in 100 µl DMEM complete media in a 96-well assay plate. The cells were incubated in a cell culture incubator for 4 h. The medium was removed and replaced with 20 µl of Opti-MEM containing PCSK9-SmBiT (final concentration of 0.8 µg/ml), followed by the addition of either 20 µl of Opti-MEM or 20 µl of Opti-MEM, containing alirocumab (final concentration of 2 µM). Next, the Nano-Glo Live Cell Substrate was diluted 1:20 in the Live Cell Substrate dilution buffer, and 10 µl of the solution was added to the test wells. Luminescence was measured after a 1 h room temperature incubation, and *Z*′ values were calculated as has been described (18) for the inner 60 wells of the assay plate. This experiment was repeated on 3 separate days, and the values were averaged across the experiments to determine the variation of the assay (coefficient of variation). The high signal was calculated from wells that did not contain antibody, and the low signal was calculated from wells that included alirocumab.

### Bioassay characteristics

#### Percentage recovery over antibody potency range.

LgBiT-LDLR HEK293 cells were plated at 20,000 cells/well in 100 µl of DMEM complete media in a 96-well plate and incubated in a cell culture incubator for 4 h. The medium was removed and replaced with 25 µl of Opti-MEM containing PCSK9-SmBiT (final concentration of 0.8 µg/ml). Next alirocumab antibody titrations were added as 2× solutions in 25 µl Opti-MEM to give the following potencies: 50, 75, 100, 125, and 150%. The 100% potency titration started at a final concentration of 500 nM alirocumab. The titrations were prepared as 1:3 serial dilutions for 11 points plus no antibody control. The reactions were incubated at room temperature for 1 h, then 12.5 µl of a 1:20 dilution of Nano-Glo Live Cell Substrate into Live Cell Substrate dilution buffer was added. Luminescence was measured after a 10-min room temperature incubation. Data were analyzed with JMP® software (SAS Institutes, Inc.). The data were analyzed with a 4PL curve fit, and relative potencies were calculated after parallelism determination. The data were averaged over three independent experiments.

### Stability-indicating property of the binding assay

Alirocumab and evolocumab were heated at 65°C for 1, 3, or 5 days and then stored at 4°C with the unheated samples, according to manufacturer instructions. To test the activity of these antibodies, we performed the PCSK9-LDLR binding assay. LDLR-LgBiT-expressing HEK293 cells (10,000 cells/well) were plated in DMEM complete media in a 96-well plate and incubated in a cell culture incubator for 4 h. The medium was removed and replaced with 25 µl of Opti-MEM containing PCSK9-SmBiT, for a final concentration of 0.8 µg/ml. Then 25 µl of each antibody titration was added at 2× concentrations in Opti-MEM. The reaction was incubated at room temperature for 1 h, then the Nano-Glo Live Cell Substrate was diluted 1:20 in the Live Cell Substrate dilution buffer, and 12.5 µl of the solution was added to the test wells.

## RESULTS

### Assay principle

**Fig. 1.**
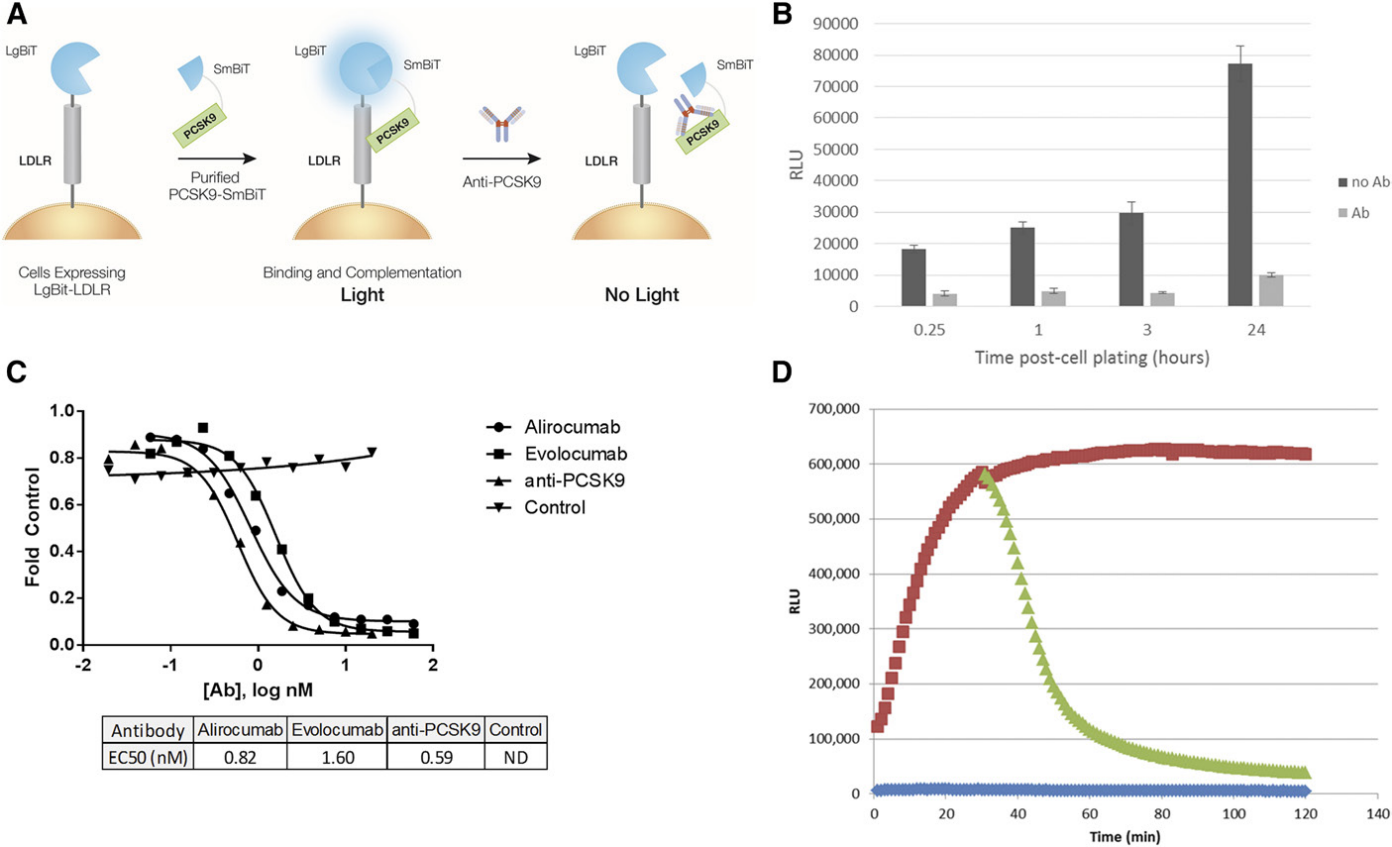
PCSK9-LDLR binding assay. A: The N-terminus of LDLR is tagged with the LgBiT protein fragment, and this protein fusion is stably expressed on the cell surface of HEK293 cells. Exogenously added PCSK9 is tagged at the C-terminus with the SmBiT protein fragment. When PCSK9 binds to LDLR, the LgBiT and SmBiT protein fragments can associate to form the active luciferase enzyme through a reversible interaction. When the interaction between LDLR and PCSK9 is inhibited (e.g., anti-PCSK9 antibody), there is no binding and therefore no luminescence. B: Frozen cells were thawed and immediately plated into assay wells. At each time point, purified PCSK9-SmBiT, media control or inhibiting antibody (alirocumab), and luciferase substrate were added. The reaction was incubated at room temperature for 15 min, and luminescence was measured. Error bars represent the standard deviations. C: PCSK9-LDLR binding assay in endpoint format. Antibody dose curves were prepared for three anti-PCSK9 antibodies (alirocumab, evolocumab, and anti-PCSK9; BPS Biosciences) and a nonspecific control antibody *(*trastuzumab). These antibody titrations were tested in the PCSK9-LDLR binding assay. Measurements were made 1 h post-antibody addition, and EC_50_ values were determined using GraphPad Prism (version 6.03). D: Real-time monitoring of PCSK9 binding to LDLR. HEK293 cells stably expressing LgBiT-LDLR were plated. The Nano-Glo substrate was added to detect the formation of active luciferase enzyme, either with PCSK9-SmBiT protein (red) or without (blue) to determine substrate-only background. At 30 min, a subset of wells was spiked with an anti-PCSK9 antibody to monitor the disruption of the PCSK9-LDLR interaction (green). RLU, relative light units.Q5

For the PCSK9-LDLR binding assay, LgBiT is fused to the N-terminus of LDLR and stably expressed in HEK293 cells. This pool of cells gives a strong assay signal that is stable beyond 20 passages (data not shown). By positioning the LgBiT at the N-terminus of LDLR, the LgBiT is extracellular. The SmBiT is fused to the C terminus of the PCSK9 protein. This fusion protein is expressed in HEK293 cells, secreted, and purified from the cell culture media. This purified PCSK9-SmBiT is added exogenously to the LgBiT-LDLR-expressing cells in media. The interaction between LDLR and PCSK9 brings LgBiT and SmBiT into close proximity, allowing complementation and formation of the active luciferase enzyme. In the presence of the NanoLuc substrate, a bright luminescence signal is generated (**Fig. 1A**). Antibodies that block the interaction between PCSK9 and LDLR (e.g., antibodies against PCSK9) result in a substantial decrease in light output. The flexible assay format allows the effect of the test candidate to be measured in endpoint format or in real time. For endpoint analysis, the luciferase substrate is added at the end of the desired incubation time, and the luminescence is measured immediately. To measure the binding of PCSK9 and LDLR in real time, we added the luciferase substrate at the same time as the reaction components, and the luminescence was measured continually.

### Assay validation

The assay can be set up in a variety of ways. The cells can be plated and incubated overnight, or they can be plated from a frozen thaw-and-use vial of cells and assayed soon after plating. This flexibility allows the user to adapt the timing of the assay for convenience. We tested how soon we could assay the binding of PCSK9 and LDLR after plating cells from a frozen thaw-and-use vial (Fig. 1B). The assay produced a robust signal within 15 min post-cell plating, and absolute signal strength continuously increased over 24 h. At each time point, an antibody that inhibits the interaction could be detected by a substantial drop in signal.

To further analyze assay set-up in the endpoint format, we analyzed the response of the assay to four antibodies, including three that are specific for the PCSK9-LDLR interaction and a nonspecific, isotype-matched control antibody (Fig. 1C). All three antibodies that bind to PCSK9 and are reported to block the interaction, including two FDA-approved drug antibodies alirocumab and evolocumab, resulted in a decrease in signal. In contrast, treatment with the control antibody (directed against Her2) had no significant effect on the signal. These data suggest that this assay specifically detects antibodies that target the interaction of PCSK9 and LDLR but not antibodies in general.

Next, we analyzed the performance of the assay in real time, which can also be performed with LgBiT-LDLR-expressing cells that are plated overnight or plated after thawing from a frozen vial. We added PCSK9-SmBiT and the luciferase substrate and immediately started measuring luminescence. The binding of these partners was rapid, as is shown by an increased signal over the substrate-only background, starting from the first reading taken at approximately 2 min post-reagent addition. The binding increased over the first 30 min. The luciferase substrate on its own showed very low background, which resulted in a large assay window [signal-to-background (S/B) = 123; signal-to-noise = 1750 at 120 min]. At 30 min, we tested the ability of this assay to respond in real time to inhibition of the interaction. When an antibody against PCSK9 was spiked into a subset of wells, there was a substantial drop in luminescence (Fig. 1D).

**Fig. 2.**
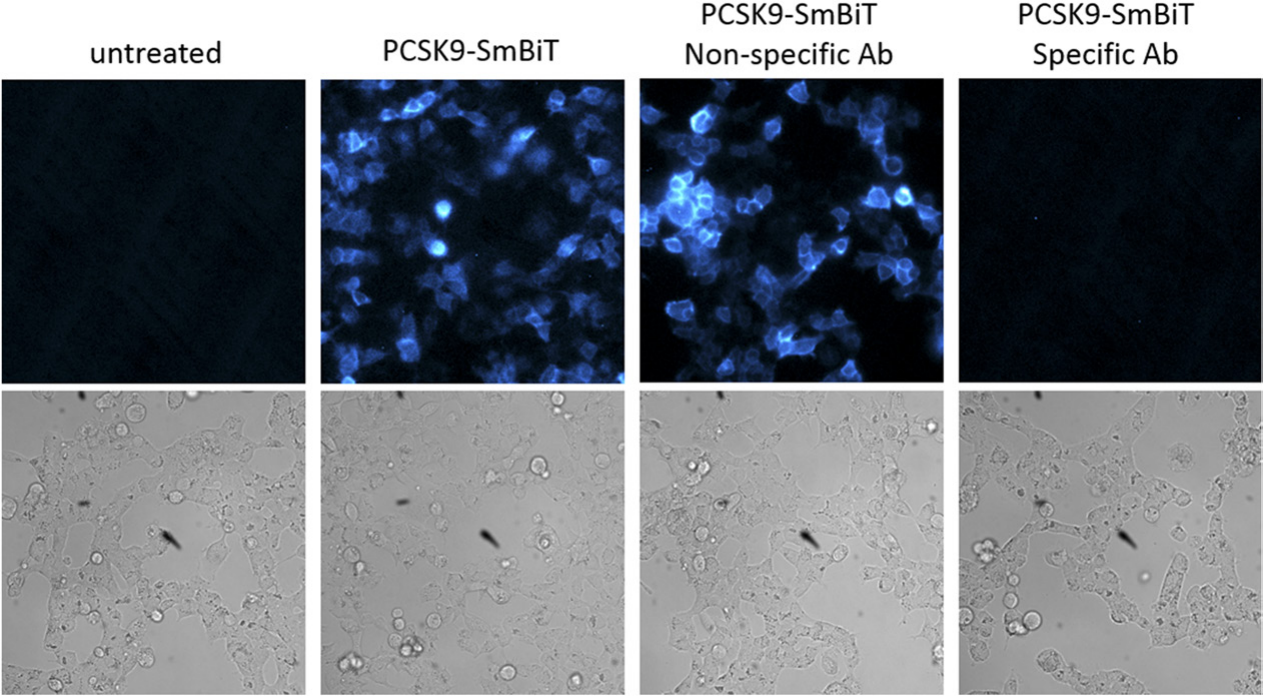
Bioluminescence imaging of the PCSK9-LDLR binding assay. The LgBiT-LDLR-expressing cells were plated on chambered coverslips. After incubation for 24 h, medium was removed, and PCSK9-SmBiT with or without antibody was added to the cells and incubated for 1 h. The PCSK9-specific antibody was anti-PCSK9 (BPS Biosciences), and the nonspecific antibody was trastuzumab. Nano-Glo substrate was added, and bioluminescence imaging performed using an Olympus LV200 bioluminescence imager.

The brightness of this binding assay allows its use in bioluminescent imaging (**Fig. 2**). LgBiT-LDLR-expressing cells were plated in chambered coverslips and treated with either PCSK9-SmBiT or a combination of PSCK9-SmBiT with either an anti-PCSK9 antibody or an isotype-matched control antibody. In the absence of PCSK9-SmBiT, no luminescent signal was detectable, as was anticipated. In contrast, addition of PCSK9 led to the generation of luminescence at the cell membrane, which is consistent with the binding of PCSK9-SmBiT to LgBiT-LDLR and the subsequent complementation between the LgBiT and SmBiT. Coincubation with a PCSK9-specific antibody disrupted this interaction, as is evidenced by the lack of bioluminescence, whereas addition of a nonspecific control antibody showed no significant effect on the binding of PCSK9-SmBiT to LgBiT-LDLR.

### Analysis of the binding event

**Fig. 3.**
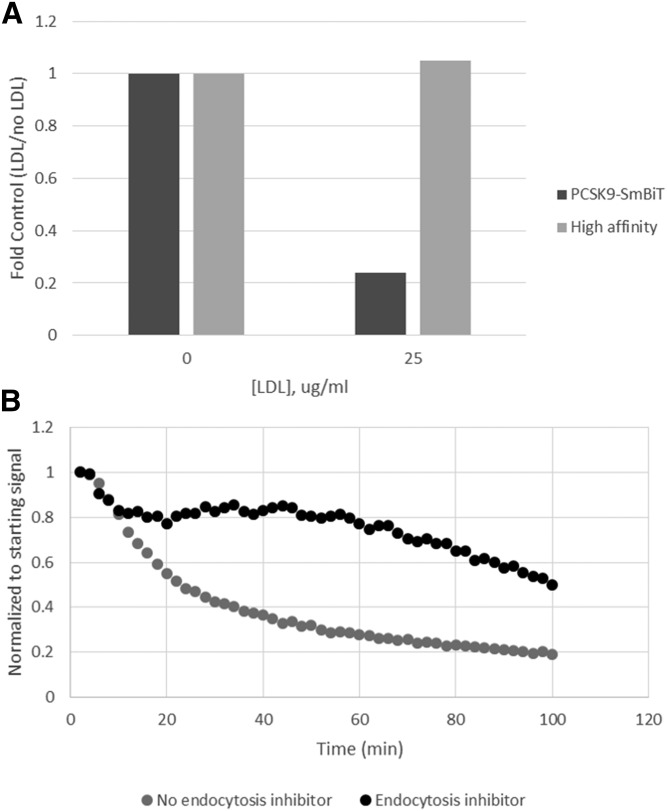
Analysis of the PCSK9-LDLR binding event. A: LgBiT-LDLR-expressing HEK293 cells were plated and incubated in the presence and absence of LDL and either PCSK9-SmBiT or a high-affinity complementation peptide. Luminescence was measured after a 45-min incubation, and data were analyzed by comparing the signal with LDL versus the signal without LDL. B: PCSK9 was added to LgBiT-LDLR cells at 4°C and incubated for 1 h. Wells were washed, and endocytosis inhibitor was added to half of the wells. The luminescence was monitored over time to determine the effect of the endocytosis inhibitor on the complex stability.

Binding of LDL and recycling of the receptor plays an important biological role in the regulation of circulating LDL. We questioned how LDL affects the binding of LDLR and PCSK9. Although LDL does not sterically hinder the binding of PCSK9 to LDLR, it nonetheless inhibits the interaction between these proteins. This inhibition is potentially through the interaction of ApoB100 on the LDL particle and PCSK9 in circulation (19–21). We performed the PCSK9-LDLR binding assay in the presence or absence of LDL (**Fig. 3A**). The signal from the binding assay decreased with the addition of LDL, suggesting that LDL either inhibits the binding of PCSK9 to LDLR or interferes with the complementation of the luciferase protein domains. In parallel, we tested a high-affinity protein complementation peptide [peptide 86 (15)] that spontaneously binds to LgBiT to generate an active luciferase enzyme. The complementation of LgBiT-LDLR and the high-affinity peptide was unaffected by the presence of LDL, suggesting that the LgBiT is still accessible for complementation.

The binding of PCSK9 to LDLR leads to internalization of the complex and eventual degradation of both PCSK9 and LDLR (4). We tested whether our assay would detect a decrease in signal over time (Fig. 3B). Cells were preloaded with PCSK9-SmBiT at 4°C to allow binding but slow internalization of the complex. After incubation, the cells were washed to remove excess PCSK9 and exchanged to assay medium that either contained an endocytosis inhibitor or did not. The plate was moved to room temperature to allow endocytosis and internalization of the complex to progress. The wells that did not have an endocytosis inhibitor showed a constant decrease in signal over time, which is consistent with complex formation followed by degradation or complex dissociation in the early endosome. The wells that contained the endocytosis inhibitor showed substantially reduced signal decay compared with the untreated sample, suggesting that endocytosis plays a role in the decrease of the signal.

### Determine feasibility for high-throughput screening

The PCSK9-LDLR binding assay was analyzed for its utility as a screening assay by determining the *Z′* value (18) and S/B on 3 separate days and comparing the variation in *Z′* and S/B across these independent experiments (**Table 1**). In addition to being a simple, homogeneous add-and-read format with no wash steps and amenability to 96-well and 384-well formats (data not shown), the assay demonstrated robustness and reproducibility. The assay window was very consistent at approximately 13 when comparing the high and low signals. The *Z′* value was also reproducible and averaged 0.83, which demonstrates the robustness of this assay for screening. The consistency of these measurements led to very low coefficients of variation at 2% and 3% for S/B and *Z′*, respectively. The robustness and reproducibility of this assay suggest that it will perform well in screening applications.

**TABLE 1.** Feasibility for high-throughput screening

	S/B	*Z*′
Day 1	13.76	0.83
Day 2	13.29	0.80
Day 3	13.27	0.85

The S/B and *Z′* values were determined by analyzing the signal from assay wells containing the LgBiT-LDLR HEK293 cells in the presence of PCSK9-SmBiT with or without anti-PCSK9 antibody. The experiment was performed on 3 separate days.

### Bioassay characteristics

#### Assessment of assay precision.

**Fig. 4.**
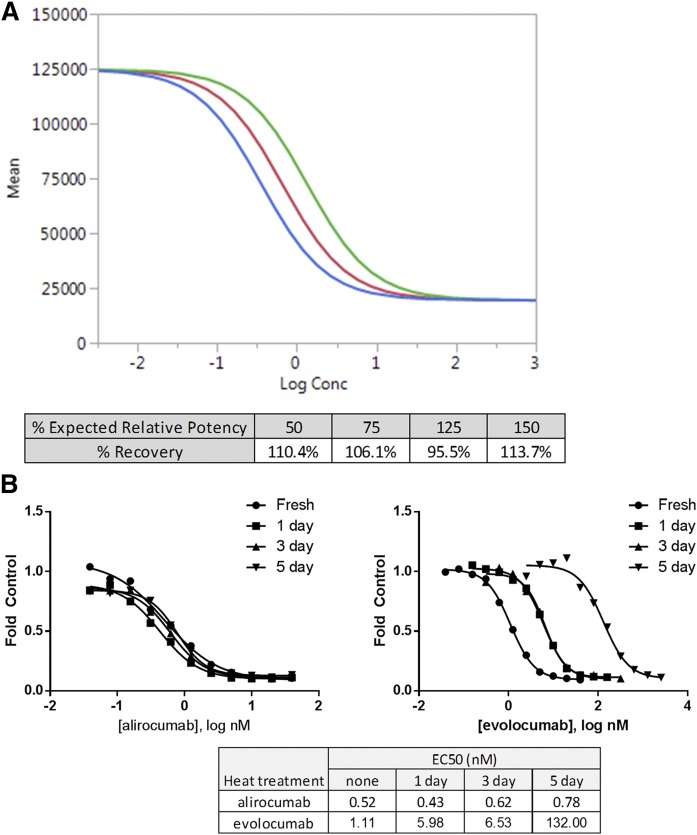
Bioassay characteristics. A: Accurate determination of potency range. Antibody titrations were prepared across the 50%–150% potency range. Data were analyzed using JMP® software (SAS Institutes, Inc.). The data were analyzed using a 4PL curve fit, and relative potencies were calculated after parallelism determination. The table shows the average data from three independent experiments. The graph shows a representative curve fit from one of the experiments comparing 100% (red) with 50% (green) and 150% (blue). B: Stability-indicating property of the PCSK9-LDLR binding assay. Alirocumab and evolocumab were heated at 65°C for various time points up to 5 days. These antibody samples were compared with the unheated antibody in the PCSK9-LDLR binding assay. EC_50_ values were determined using GraphPad Prism (version 6.03). LogConc, log concentration (nM).

To determine whether the assay can accurately report EC_50_ values, we prepared antibody titrations across a 50%–150% potency range. Dilution ranges were selected to obtain good coverage at both upper and lower asymptotes as well as provide sufficient data points to reliably determine EC_50_. A series of theoretical potency samples (50, 75, 125, and 150%) were prepared on 3 separate days. The EC_50_ values obtained for each set closely match the expected EC_50_ values on the basis of the theoretical potencies, which indicates that the assay is sensitive enough to distinguish among the subtle changes in potency and well within the 70%–130% recovery-approved guideline (ICH Guideline Q2[R1]) (**Fig. 4A**).

### Evaluation of stability-indicating property of the assay

To determine whether the PCSK9-LDLR binding assay provides sufficient sensitivity to detect changes in potency due to heat stress, we heated alirocumab and evolocumab and analyzed their activity compared with control (unheated) antibody (Fig. 4B). Alirocumab showed no difference among the unheated and heated samples, demonstrating that this antibody is stable at the tested temperature. In contrast, evolocumab showed a substantial drop in activity after heating, as was shown by the time-dependent right shift in EC_50_. These results indicate that the PCSK9-LDLR binding assay can detect loss of antibody activity due to heat stress and could potentially be used in stability studies for therapeutic antibody drug development.

## DISCUSSION

Innovative methods to analyze real-time binding of a full-length receptor to its binding partners are needed because of the importance of these binding events. In this study, we analyzed the interaction between PCSK9 and LDLR, because the importance of this interaction in cholesterol metabolism is becoming more evident, and the early clinical therapeutics are showing great promise. Current assay technologies that are set-up in an artificial, biochemical environment involving interrogation of fragments of the binding partners have limited utility. In contrast, the novel assay we described analyzes the binding of PCSK9 and LDLR in the more natural, cell-based environment with full-length LDLR expressed on the cell surface and soluble, full-length PCSK9 in the cell culture media. Each protein is tagged with a peptide. Other groups have adapted LDL uptake assays to screen for PCSK9 modulators, but these assays lack the robustness and sensitivity of the PCSK9-LDLR binding assay (22). The binding assay described here is the first plate-based, cell-based assay that directly analyzes the PCSK9-LDLR interaction.

The assay is based on protein complementation technology, and we show that this allows detection of the interaction both in endpoint and in real time (Fig. 1B–D). The detectable binding of PCSK9 and LDLR in this assay system as well as the perturbation by anti-PCSK9 antibodies suggest that the small protein fragments (1 and 18 kDa) that are attached to each protein of interest do not change the binding or function. The specificity for antibodies that are known to block this interaction also indicates that luciferase complementation depends on the binding of the two proteins of interest and not the luciferase protein fragments. The nonspecific, isotype-matched antibody does not affect the interaction, suggesting that antibodies in general will not disrupt the complementation of the luciferase fragments, but it depends on the PCSK9 and LDLR interaction to bring them together. The bioluminescent signal generated is also sufficiently bright to be detected by imaging (Fig. 2). This provides another way to analyze the binding and specific disruption of this interaction and allowed us to validate proper localization of the complex. Additionally, this assay could be used for further studies that investigate in more detail the localization, subsequent internalization, and degradation of this complex.

The assay reflected the expected biology by showing a decrease in binding in the presence of LDL (Fig. 3A). The signal also decreased when excess PCSK9 was removed (Fig. 3B). This decrease was reversed in the presence of an endocytosis inhibitor. Because this binding assay is cell based and does not have the limitations of a biochemical assay with protein fragments, there are many additional experiments that can be done to interrogate the biology (e.g., analysis of binding/internalization kinetics, localization of complex, effect of various cellular factors on this interaction).

The flexibility and ease-of-use of the assay fits well for high-throughput screening applications. The flexibility allows assay set-up in 96-well or 384-well formats plus the ability to monitor drug candidate-induced changes at a particular endpoint or kinetically over time. The assay performed well in *Z′* determinations over multiple days, suggesting that it is sufficiently robust for screening applications (Table 1). There are currently no assays available to screen for new drug candidates that inhibit the PCSK9 and LDLR interaction in a cell-based assay utilizing full-length proteins. This assay provides an opportunity to test for more biologically relevant inhibitors.

The accuracy of the assay is also important for ensuring that the assay can distinguish among samples with different potencies. The assay was capable of reporting the appropriate EC_50_ values when comparing antibody titrations that were across a tight range (50%–150%) (Fig. 4A). This assay meets important requirements for a potency bioassay, in particular, high precision and low CV. The assay was also sensitive enough to detect changes in potency after heat stress (Fig. 4B). One antibody (alirocumab) was clearly more resistant to heat stress, showing no change in activity, compared with evolocumab, which showed a drastic change after only 1 day. Antibody development and manufacturing routinely include antibody stability testing, which requires suitable assays that can reliably measure changes in antibody potency. We demonstrate that the PCSK9-LDLR binding assay can distinguish between antibodies with differential susceptibility to heat stress.

The PCSK9-LDLR binding assay provides an example on using the NanoBiT complementation technology for the development of receptor-ligand binding assays. By tagging the extracellular region of a receptor on the cell surface with a small, NanoLuc protein fragment, we can interrogate the binding of multiple extracellular binding partners, including proteins (such as PCSK9), chemical ligands, or small-molecule agonists or antagonists. Protein fusions can be created with the complementary NanoLuc peptide fragment, then purified and added to the cell culture media. Similarly, small-molecule compounds can be fused to the peptide and added extracellularly. This assay concept fills a need for a more biologically relevant assay to analyze receptor-ligand binding kinetics.
